# Redefining early stage nonsmall cell lung cancer: The dawn of the risk‐adaptive and biologically precise era

**DOI:** 10.3322/caac.70102

**Published:** 2026-09-09

**Authors:** Clarissa Mathias, Fabio Ynoe Moraes

**Affiliations:** ^1^ Oncoclinicas and Cancer Center Hospital Santa Izabel Oncoclinicas Salvador Bahia Brazil; ^2^ Kingston Health Sciences Center Sinclair Cancer Research Institute Queen’s University Kingston Ontario Canada

For decades, the management of early stage nonsmall cell lung cancer (NSCLC) was guided primarily by anatomic staging and surgical resection. Although this approach has achieved favorable outcomes for many patients, it does not fully account for the biologic heterogeneity that influences recurrence risk and treatment response. Contemporary advances in lung cancer screening, surgical techniques, precision radiotherapy, molecular profiling, and perioperative systemic therapies have collectively transformed the therapeutic landscape, supporting a more risk‐adaptive approach that integrates anatomic, biologic, and patient‐specific factors into clinical decision‐making.

In this context, the review by Zhang et al.^1^ provides a comprehensive overview of the advances that are reshaping the management of localized NSCLC. Progress in lung cancer screening, minimally invasive and parenchyma‐sparing surgery, stereotactic ablative radiotherapy (SABR) and other highly conformal radiotherapy techniques, together with the integration of perioperative immunotherapy and molecularly targeted therapies, has broadened the therapeutic landscape and enabled increasingly individualized treatment approaches.

Importantly, the review highlights the evolving limitations of relying solely on the tumor‐node‐metastasis classification to guide treatment decisions. Although tumor‐node‐metastasis staging remains the cornerstone of prognostic assessment and therapeutic planning, it provides limited information regarding tumor biology, host immune interactions, or the likelihood of micrometastatic disease.^2^ The increasing availability of molecular profiling, circulating tumor DNA analysis, and other emerging biomarkers offers opportunities to complement anatomic staging with biologic risk assessment, potentially enabling more individualized treatment strategies. Rather than replacing established staging systems, these advances support a more integrated framework in which anatomic extent of disease, molecular alterations, immune characteristics, and dynamic biomarkers collectively inform clinical decision‐making. As the field continues to evolve, the central challenge will be determining how these emerging tools can be incorporated into evidence‐based care while maintaining equitable access across diverse health care settings.

## THE SCREENING REVOLUTION AND THE CHANGING EPIDEMIOLOGICAL LANDSCAPE

The widespread implementation of low‐dose computed tomography screening has fundamentally altered the presentation of NSCLC. Landmark trials, specifically the National Lung Screening Trial^3^ and the NELSON trial,^4^ have demonstrated significant mortality reductions, cementing low‐dose computed tomography as the gold standard for high‐risk populations. However, this screening success has precipitated a *stage migration*, characterized by a dramatic increase in the detection of early stage lesions, particularly ground‐glass opacities.

Simultaneously, the epidemiology of lung cancer is evolving. We are observing a rising incidence of NSCLC among younger individuals, females, and never‐smokers, particularly in East Asian cohorts. These patients often present with indolent, ground‐glass opacity–dominant adenocarcinomas driven by specific oncogenic mutations. The clinical challenge now lies in distinguishing these indolent lesions from aggressive, invasive cancers. Overtreatment of a stable ground‐glass opacity can lead to unnecessary surgical morbidity, whereas undertreatment of a biologically aggressive early stage lesion risks systemic relapse. This necessitates the development of sophisticated risk‐stratification tools that integrate radiomics and molecular biomarkers to guide the timing and extent of intervention.

## SURGICAL EVOLUTION: FROM MAXIMALLY TOLERABLE TO MINIMALLY INVASIVE AND PARENCHYMA‐SPARING

The surgical management of early stage NSCLC is undergoing its most significant refinement since the 1960s. The historical preference for lobectomy, established by the Lung Cancer Study Group in 1995, is being challenged by high‐quality evidence supporting parenchyma‐sparing resections. The results of the JCOG0802/WJOG4607L and CALGB 140503 phase 3 trials have been transformative.^5^, ^6^ These studies demonstrated that, for peripheral tumors ≤2 cm with a low consolidation‐to‐tumor ratio, segmentectomy is noninferior to lobectomy in terms of disease‐free survival and, in the case of JCOG0802, superior in terms of overall survival.

This shift toward segmentectomy and wedge resection is not merely about reducing the volume of lung tissue removed; it is about surgical precision integrated with biologic understanding. By preserving pulmonary function without compromising oncologic outcomes, we improve the patient's quality of life and maintain their physiologic reserve for potential future treatments. The modern thoracic surgeon must now be adept at navigating complex segmental anatomy, often aided by three‐dimensional reconstruction and robotic platforms, to deliver a tailored resection that matches the tumor's biologic risk profile.^6^

## LOCAL THERAPY BEYOND SURGERY: THE EXPANDING ROLE OF SABR

The evolution of local therapy exemplifies the broader transition toward risk‐adaptive management in early stage NSCLC. Surgery remains the preferred treatment for medically operable patients, whereas SABR has become the standard of care for those who are medically inoperable, achieving excellent local control with minimal treatment‐related morbidity. Current clinical guidelines increasingly emphasize multidisciplinary evaluation to individualize treatment selection according to tumor characteristics, physiologic reserve, comorbidities, and patient preferences rather than relying solely on operability.

Importantly, the discussion is gradually shifting beyond the traditional debate of surgery versus SABR. Although randomized evidence comparing these modalities in operable patients remains limited, available data suggest that the optimal local treatment is unlikely to be determined by procedural superiority alone.^7^ Instead, treatment decisions will increasingly depend on integrating anatomic staging with biologic determinants of recurrence risk and treatment response.

Emerging technologies, including radiomics, functional imaging, circulating tumor DNA, and other molecular biomarkers, offer the potential to refine patient selection and identify individuals who are most likely to benefit from treatment intensification or de‐escalation. Likewise, growing interest in combining SABR with systemic immunotherapy reflects the recognition that local treatment may contribute not only to durable local control but also to systemic antitumor immunity. As highlighted by Zhang and colleagues, the future of local therapy lies not in choosing between competing modalities but in matching the right treatment to the right patient through increasingly precise biologic and clinical risk stratification.

## THE PERIOPERATIVE IMMUNOTHERAPY EXPLOSION

Perhaps the most radical change in the management of resectable NSCLC is the rapid integration of immune checkpoint inhibitors. The neoadjuvant landscape was redefined by the CheckMate 816 trial,^8^ which demonstrated that adding nivolumab to platinum‐doublet chemotherapy significantly increased the pathologic complete response rate (24.0% vs. 2.2%) and improved event‐free survival. This success has been followed by *dual‐platform* or *sandwich* approaches, in which immunotherapy is administered both before and after surgery.

Trials such as KEYNOTE‐671 (pembrolizumab),^9^ AEGEAN (durvalumab),^10^ and Neotorch (toripalimab)^11^ have consistently demonstrated that perioperative immunotherapy provides a robust survival advantage. These findings have elevated pathologic and major pathologic complete responses as critical surrogate end points for long‐term cure. However, this explosion of data brings new clinical dilemmas. We must now determine which patients benefit most from neoadjuvant induction versus those who require adjuvant consolidation.^12^, ^13^ Furthermore, the management of patients who do not achieve a pathologic complete response—those with *resistant* biology—remains a frontier for therapeutic escalation and novel combination strategies.

## ADJUVANT TARGETED THERAPIES: TRANSLATING METASTATIC SUCCESS TO CURATIVE SETTINGS

Although immunotherapy has revolutionized the treatment of *cold* tumors, oncogene‐directed therapies have redefined the standard for patients with specific molecular drivers. The ADAURA^14^, ^15^ trial was a watershed moment, demonstrating that adjuvant osimertinib provides an unprecedented disease‐free and overall survival benefit for patients with resected EGFR‐mutant (exon 19 deletion or L858R) stage IB–IIIA NSCLC. Similarly, the ALINA^16^ trial recently demonstrated that adjuvant alectinib significantly improves DFS in ALK‐positive patients compared with chemotherapy.

These results mandate a change in clinical workflow. Reflex molecular testing for at least EGFR and ALK is no longer optional for resected stage IB–IIIA NSCLC; it is a clinical imperative. Omitting these tests denies patients access to highly effective, well tolerated therapies that significantly increase the probability of a permanent cure. The transition from treating metastatic disease to treating micrometastatic disease with targeted agents represents the pinnacle of biologic precision in the current era.

## THE NEXT FRONTIER: LIQUID BIOPSIES, MINIMAL RESIDUAL DISEASE, AND MULTI‐OMICS

The future of risk‐adaptive management lies in our ability to monitor disease in real time. The detection of circulating tumor DNA and minimal residual disease (MRD) is poised to replace *watchful waiting* with *molecular surveillance*. Insights from the TRACERx study^17^ have shown that postoperative circulating tumor DNA detection can predict radiologic recurrence by a median of several months, providing a window for early intervention.

We envision a future in which clinical trials are designed around MRD status. In this model, adjuvant therapy could be escalated for MRD‐positive patients who are at the highest risk of relapse, whereas therapy could be safely de‐escalated or omitted for MRD‐negative patients, sparing them from unnecessary toxicities and financial burden. This transition from anatomic staging to biologic monitoring will allow us to treat the *molecular relapse* rather than waiting for the *radiologic recurrence*.

## BRIDGING THE GAP: GLOBAL DISPARITIES AND HEALTH EQUITY

The implementation of this risk‐adaptive paradigm is highly unequal. Access to low‐dose computed tomography screening, next‐generation sequencing, robotic surgery, immune checkpoint inhibitors, and tyrosine kinase inhibitors is concentrated in high‐income countries. In many regions, lung cancer is still diagnosed at advanced stages, and precision tools remain out of reach. Addressing these disparities requires international collaboration to reduce costs of diagnostic platforms and therapeutics, along with policy reforms integrating screening into public health systems. A risk‐adaptive paradigm available only to the affluent is a partial victory; biologic precision must become the global standard of care.

## CONCLUSION

The management of early stage NSCLC has entered a transformative era, moving beyond the limitations of the past toward biologic precision. This framework integrates early detection through screening, parenchyma‐sparing surgical excellence, perioperative immunotherapy, and targeted agents. The path forward requires multidisciplinary tumor boards to use molecular and radiologic data for personalized, risk‐adaptive treatment plans, maximizing cure rates while minimizing treatment burden worldwide.

## CONFLICT OF INTEREST STATEMENT

Fabio Ynoe Moraes reports support for professional activities from AstraZeneca outside the submitted work. Clarissa Mathias disclosed no conflicts of interest.
